# Transcriptome and Phytochemical Analysis Reveals the Alteration of Plant Hormones, Characteristic Metabolites, and Related Gene Expression in Tea (*Camellia sinensis* L.) Leaves During Withering

**DOI:** 10.3390/plants9020204

**Published:** 2020-02-06

**Authors:** Ping Xu, Hui Su, Shiqi Zhao, Rong Jin, Haiyan Cheng, Anan Xu, Wanyi Lai, Xueren Yin, Yuefei Wang

**Affiliations:** 1 Department of Tea Science, Zhejiang University, Hangzhou 310058, China; su10928@163.com (H.S.); zhaosq89@163.com (S.Z.); 21716066@zxju.edu.cn (H.C.); 21816066@zju.edu.cn (A.X.); 21816158@zju.edu.cn (W.L.); 2 Agricultural Experiment Station, Zhejiang University, Hangzhou 310058, China; rong@zju.edu.cn; 3 Department of Horticulture, Zhejiang University, Hangzhou 310058, China; xuerenyin@zju.edu.cn

**Keywords:** withering, plant hormones, abscisic acid, metabolites, theanine

## Abstract

Plant hormones play an important role in the chemical metabolism of postharvest plants. However, alterations in plant hormones of postharvest tea and their potential modulation of quality-related metabolites are unknown. In this study, the dynamic alterations of abscisic acid (ABA), salicylic acid (SA), jasmonic acid (JA), and critical metabolites, such as catechins, theanine, and caffeine, in tea leaves were analyzed during withering from 0 to 24 h. It was found that the ABA content increased from 0 to 9 h but decreased thereafter, JA continuously increased, and the SA content showed no significant change. With the exception of gallocatechin (GC) and epicatechin (EC), the amounts of other critical components were significantly reduced at 24 h. Transcriptome analysis showed that compared with 0 h, 2256, 3654, and 1275 differentially expressed genes (DEGs) were identified at 9, 15, and 24 h, respectively. For all comparisons, DEGs corresponding to the pathways of “phenylalanine, tyrosine, and tryptophan biosynthesis” and “phenylalanine metabolism”, involved in the biosynthesis of catechins, were significantly enriched. Weighted correlation network analysis (WGCNA) of co-expression genes indicated that many of the modules were only correlated with a specific trait during the withering process; the dark olive-green module, however, was correlated with two traits, ABA and theanine. Our study indicates that withering induced dramatic alterations in gene transcription as well as levels of hormones (ABA, JA, and SA) and important components, and that ABA regulated theanine metabolism during this process.

## 1. Introduction

Tea, manufactured from the leaves of *Camellia sinensis*, is the second most widely consumed beverage in the world, after water [1]. The worldwide popularity of tea is attributable not only to its distinct and pleasant flavors, but also to its health-promoting potential, both of which are due to the abundant metabolites that it contains, such as polyphenols, theanine, and caffeine [2]. While teas are traditionally classified as green tea, oolong tea, black tea, and pu-erh tea (or dark tea), the main manufacturing procedures are similar, which includes picking (plucking), withering (spreading), fixing (only for green tea), macerating, rolling, fermenting (oxidizing, not for green tea), and drying [3]. For tea production, withering is the first processing step, during which substantial changes take place with respect to its secondary metabolites, providing an important basis for the formation of a unique sensory quality [3]. For oolong tea, it has been found that the total content of glycosidically bound volatiles (GBVs) gradually increased at the withering step and then remarkably increased after the fixing step at 230 °C [4]. The dehydration stress during withering can promote the special flavors of various teas by inducing significant changes in the gene transcription and amounts of tea flavor compounds [5].

The molecular characteristics of tea withering are similar to leaf senescence, according to the results of functional annotation [6]. There are multiple significant alterations involved in the physiology and biochemistry of tea during the senescence process, such as the degradation of chlorophyll, lipids, and proteins [7]. Senescence, a biological process, is strictly regulated by numerous molecules that can activate or enhance the expression levels of related genes [8,9,10]. Previous reports have shown that plant hormones play a critical role in plant senescence [11,12]. For example, salicylic acid (SA) plays an important role in responding to biotic and abiotic stress in plants, and it is also related to plant leaf senescence [13,14,15]. The deletion mutants of SA synthesis genes or signal transduction genes (*sid2*, *eds1*, *pad4*, and *npr1*) exhibit the phenotypes of delayed leaf senescence, and SA and senescence cause a similar pattern of transcriptome changes [13]. The leaves of *Arabidopsis thaliana* treated with exogenous jasmonic acid (JA) showed premature senescence. During senescence, the expression levels of JA synthesis-related genes were upregulated, and the JA content increased. In addition, JA and methyl jasmonate (MeJA) could induce the expression levels of many senescence-related genes in leaves, such as *SENESCENCE 4* (*SEN4*) and *SEN5* [16]. Abscisic acid (ABA), an important growth inhibitor, is also involved in the regulation of plant leaf senescence. Exogenous ABA can upregulate the expression levels of senescence-related genes (*SAGs*) and promote leaf senescence [16]. The *AtNAP* transcription factor can directly act on the target gene, *SAG113*, to promote the synthesis of ABA and induce leaf senescence [16].

While there have been a number of studies on tea withering (senescence), less attention has been paid to hormone changes during the withering process and their potential regulation of the metabolism of other substances. Therefore, in the present work, we measured the dynamic changes of the main senescence-related hormones (SA, JA, and ABA) and crucial components in tea leaves harvested over a 24 h period to investigate the relationship between critical components (catechins, theanine, and caffeine) and hormones (ABA, SA, and JA). We found that ABA acted at the transcription level to influence the theanine content during withering.

## 2. Results and Discussion

### 2.1. Changes in Hormone Content of Tea Leaves During the Withering Process

**Figure 1:**
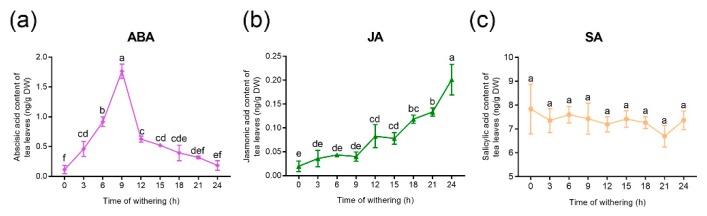
Content changes of abscisic acid (ABA) (**a**), jasmonic acid (JA) (**b**), and salicylic acid (SA) (**c**) at different timepoints during the withering process. Labeled points (not connected by the same letter) are significantly different at *p* value < 0.05, according to Duncan’s test.

Withering is a process of senescence that affect the flavor and quality of tea during manufacturing. Hormones such as ABA, SA, and JA play an important role in regulating plant senescence. Therefore, we hypothesized that hormones are related to withering. The amounts of hormones (ABA, SA, and JA) were measured in tea that had withered for 0, 3, 6, 9, 12, 15, 18, and 24 h (Figure 1). The ABA content increased from 0 (average of 0.115 ng/g) to 9 h (average of 1.766 ng/g) and decreased thereafter (Figure 1a). With the extension of withering time, the content of JA increased gradually, and the maximum content was observed at 24 h (average of 0.201 ng/g) (Figure 1b). By contrast, there was only a slight change in SA concentration during the withering process (Figure 1c). These findings indicate that ABA and JA may be involved in withering, while SA may not play an important role in withering.

### 2.2. Determination of Catechins, Free Amino Acids, and Purine Alkaloids

**Figure 2:**
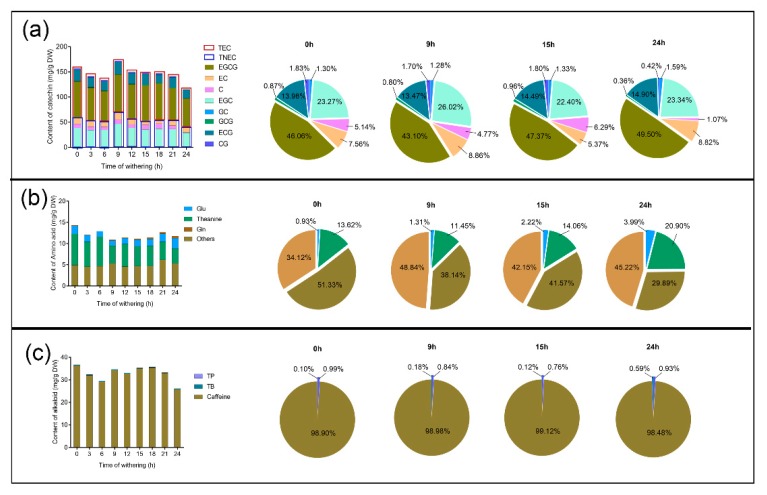
Changes in the content and proportion of catechins (**a**), theanine (**b**), and caffeine (**c**) at different stages of the withering process. The total height of each column and the total area of each pie chart both indicate the total amount of 8 catechins, all amino acids, including theanine, and all alkaloids, including caffeine. C, catechin; CG, catechin gallate; EC, epicatechin; ECG, epicatechin gallate; EGC, epigallocatechin; GC, gallocatechin; GCG, gallocatechin gallate; ECGC, epigallocatechin gallate; TEC, total content of ester catechins; TNEC, total content of non-ester catechins; Glu, glutamate; Gln, glutamine; TB, theobromine; TP, theophylline.

In tea, nearly 4000 bioactive compounds have been identified [17], in which catechins, purine alkaloids, and free amino acids are the most important compounds [18]. They are beneficial to the formation of tea flavor and human health through, for example, preventing cardiovascular diseases [19], lowering blood pressure [20], and assisting weight loss [21]. Therefore, dynamic changes of these compounds in tea leaves were identified during the withering process at different timepoints (Figure 2). The maximum amounts of ester catechins and non-ester catechins were observed at 9 h, and the total content of catechins decreased at 24 h (average of 115.042 mg/g) (Figure 2a). The amounts of catechin (C), gallocatechin gallate (GCG), epigallocatechin gallate (ECGC), and catechin gallate (CG) were significantly decreased (Figure S1). Since the highest ABA content was observed at 9 h (Figure 1a), four timepoints, 0, 9, 15, and 24 h, were selected to examine the proportion of catechin, caffeine, and theanine. While the percentage of EGCG in all catechins increased from 0 (46.06%) to 3 h (49.50%), the EGCG content decreased (Figure 2a, Figure S1). At all of the assayed timepoints of the withering process, theanine, glutamate (Glu), and glutamine (Gln) were the main amino acids in tea leaves (Figure 2b), and the content of theanine decreased gradually with withering time (Figure S2a). Alkaloids, theobromine (TB), theophylline (TP), and caffeine were measured, whereby caffeine was the main component and decreased significantly at 24 h (Figure 2c, Figure S2b). These results indicate that the amount of these critical components in tea were affected, to varying degrees, during the withering process, which is consistent with the results of previous studies [22,23,24].

### 2.3. Global Transcriptome Analysis of Tea Leaves During the Withering Process

**Figure 3:**
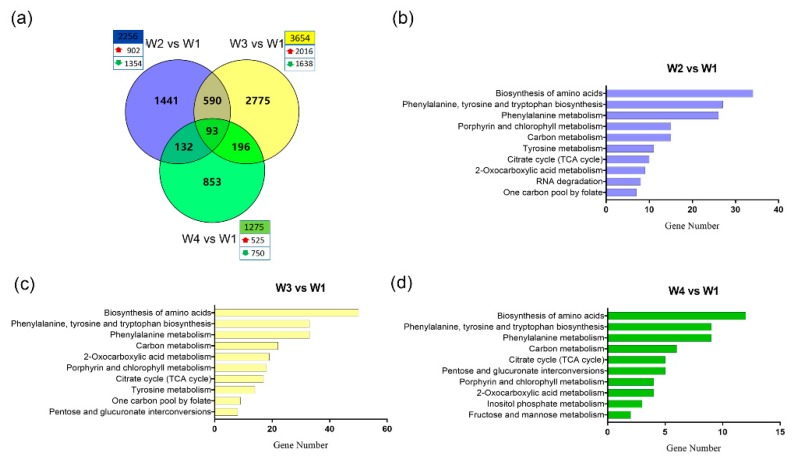
The numbers of differentially expressed genes (DEGs) and significantly enriched Kyoto Encyclopedia of Genes and Genomes (KEGG) pathways. (**a**) Venn diagram showing the share and specific DEG numbers in different combinations, displayed in the overlapping and non-overlapping regions, respectively. The total numbers of DEGs in W2 vs. W1 (blue circle), W3 vs. W1 (yellow circle), and W4 vs. W1 (green circle) are shown in the first line of the box, including the numbers of upregulated (upward red arrow) and downregulated (downward green arrow) DEGs. Histogram showing the top 10 (ranked by gene number) enriched KEGG pathways in W2 vs. W1 (**b**), W3 vs. W1 (**c**), and W4 vs. W1 (**d**).

The total RNA from tea leaves during withering for 0 (W1), 9 (W2), 15 (W3), and 24 h (W4) was analyzed by performing RNA-seq experiments. Three independent biological replicates were analyzed for each condition (12 samples in total). Approximately 0.77 billion high-quality reads (average: ~65 million reads from each sample) (Q30 > 93%) were generated for all of the tea samples (Table S1). TopHat was used to map all clean reads to the *C. sinensis* genome (cv. Shuchazao). Finally, a total of 78,532 genes, including 52,332 known (Table S2) and 26,200 novel genes, were generated from the RNA-seq data. The uniquely mapped reads from each sample were processed using StringTie to determine the normalized expression level as fragments per kilobase of the transcript length per million mapped reads (FPKM) of each transcript. The differentially expressed genes (DEGs) (|log2 (fold change)| ≥ 2 and *p* value < 0.05) of different withering groups (W2, W3, and W4) and the control group (W1) were generated using Ballgown. There were 2256 (up: 902; down: 1354), 3654 (up: 2016; down: 1638), and 1275 (up: 525; down: 750) DEGs generated from W2 vs. W1, W3 vs. W1, and W4 vs. W1, respectively (Figure 3a, Table S3), which indicated that these DEGs continued to function during the withering process for 24 h. Hormones and critical components might cause significant changes in the expression levels of these DEGs.

The DEGs of all comparison groups were subjected to Kyoto Encyclopedia of Genes and Genomes (KEGG) pathway enrichment analysis, and the top 10 (ranked by gene number) pathways are shown (Figure 3b–d). There were seven shared pathways significantly enriched in the three comparison groups. The shared pathways, “phenylalanine, tyrosine, and tryptophan biosynthesis”, and “phenylalanine metabolism” are related to the biosynthesis of catechins. “The biosynthesis of amino acids” was also enriched significantly in the DEGs of the three comparison groups and was fairly active in the withered tea leaves. In addition, the DEGs of two comparisons (W2 vs. W1 and W3 vs. W1) were involved in “purine metabolism” (Table S4), which was related to the accumulation of caffeine. These results indicated that significant changes of the gene expression levels cause a dramatic alteration in the amount and proportions of catechins, free amino acids, and caffeine.

### 2.4. Construction of a Gene Co-Expression Network and Identification of Content-Related Modules

During the withering process, the amounts of hormones (ABA and JA) and critical components (catechins, theanine, and caffeine) in tea leaves were dramatically altered (Figure 1 and Figure 2). We calculated the correlation coefficient between the change patterns in both hormones and critical components (Figure S3). JA was negatively correlated with all critical components. ABA had a slight positive correlation with the critical components in tea leaves during the withering process, with the exception of theanine (Figure S3). There was a positive correlation between SA and theanine (Figure S3). These results suggest that hormones and critical components may interact with each other in tea leaves during the withering process. To further investigate the relationship between hormones and critical components in terms of the level of transcription, we performed weighted correlation network analysis (WGCNA) to construct a co-expression network of co-expressed genes (average FPKM > 5 of all samples) (Figure S4). Since the changes in the SA, gallocatechin (EC), and gallocatechin (GC) amounts were not significant at the four timepoints, we used the amounts of hormones (JA and ABA) and critical components (theanine, caffeine, epigallocatechin (EGC), C, EGCG, GCG, epicatechin (ECG), and CG) to construct a co-expression network. The analysis generated 10 modules, and six modules exhibited a strong correlation (*r* ≥ 0.75, *p* value < 0.05) between the gene expression and substance accumulation pattern. The royal-blue module showed a relatively higher correlation (*r* = 0.97, *p* value = 4e-8) with JA. ABA was correlated with the dark olive-green (*r* = 0.8, *p* value = 0.002), dark red (*r* = 0.92, *p* value = 2e-5), and dark sea-green (*r* = 0.82, *p* value = 0.001) modules. Theanine was correlated with the dark olive-green (*r* = 0.75, *p* value = 0.005) and blue modules (*r* = 0.84, *p* value = 5e-4). There were two modules associated with caffeine and catechins, the blue and white modules (*r* ≥ 0.75, *p* value < 0.05) (Figure S4). The dark olive-green module was correlated with ABA and theanine, indicating that the co-expressed genes in the module may be related to alterations in ABA and theanine in tea leaves during the withering process.

**Figure 4:**
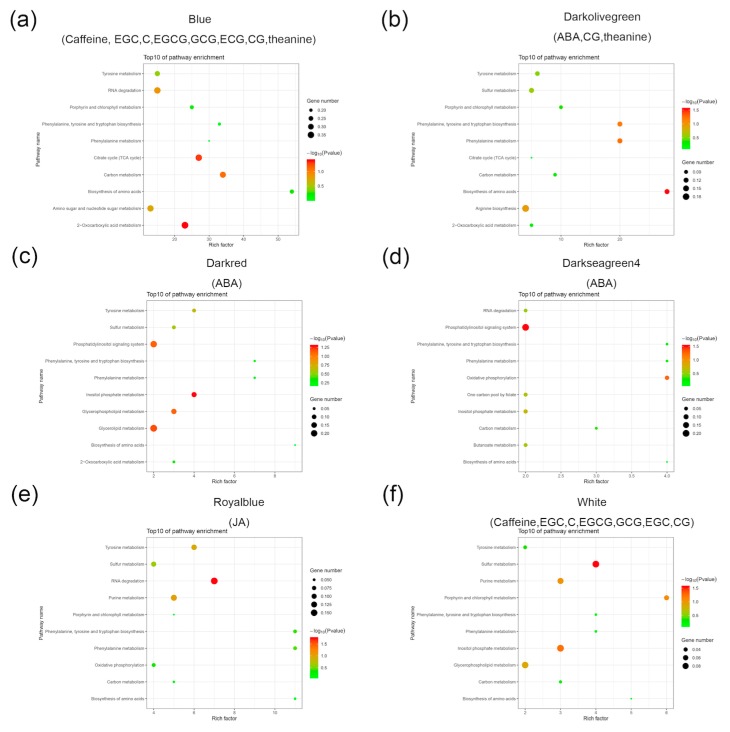
Bubble chart shows the top 10 (ranked by gene number) KEGG pathways in six modules ((**a**): Blue module, (**b**): Darkolivegreen module, (**c**): Daekred module, (**d**): Darkseagreen4 module, (**e**): Royalblue module, (**f**): White module) associated with caffeine and catechins. The X-axis indicates the “rich factor”, represented by the ratio of co-expressed gene numbers to the total gene numbers of each pathway, and the left Y-axis represents the KEGG pathways. Low *p* values are shown in red, and high *p* values are shown in green on the right. The area in the circle represents the co-expressed gene number.

KEGG pathway enrichment analysis of the modules indicated a correlation between hormones and critical components (*r* ≥ 0.75, *p* value < 0.05) and highlighted key representative biological functions of the co-expressed genes (Table S5). The top 10 (ranked by gene number) pathways are shown (Figure 4). In the dark olive-green module, the pathways, “biosynthesis of amino acids”, “phenylalanine metabolism”, and “phenylalanine, tyrosine, and tryptophan biosynthesis”, were significantly enriched, indicating that these pathways may be involved in the changes of ABA and theanine in tea during the withering process (Figure 4, Table S5). In the blue and white modules, “biosynthesis of amino acids”, “carbon metabolism”, “phenylalanine metabolism”, “porphyrin and chlorophyll metabolism”, “porphyrin and chlorophyll metabolism”, and “tyrosine metabolism” were significantly enriched (Figure 4, Table S5), indicating that these pathways play an important role in the regulation mechanism of the alteration in critical components in tea leaves during the withering process. These results indicated that the changes of hormones and critical components in tea leaves during the withering process were regulated by a variety of co-expressed genes. ABA and theanine interacted with each other in the co-expressed genes of the dark olive-green module.

### 2.5. Effect of Withering on Catechins, Theanine, and Caffeine Metabolism

**Figure 5:**
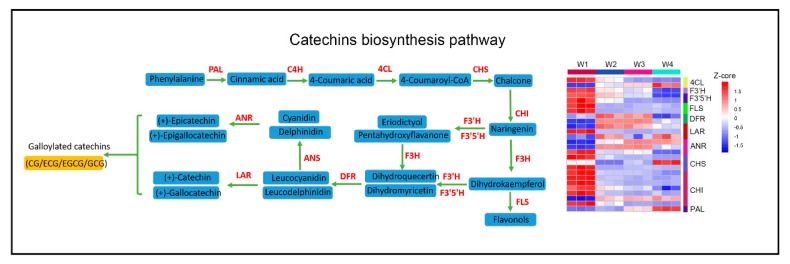
Effect of withering on the catechin biosynthesis pathway in tea leaves. A simplified model of the catechin biosynthesis pathway in tea plants (left) and a heat map, showing the expression pattern of related DEGs based on the Z-core value at different timepoints during the withering process. The DEGs were strictly filtrated by |log2 (fold change)| ≥ 2 and *p* value < 0.05, with at least one of three comparisons (W2 vs. W1, W3 vs. W1, and W4 vs. W1). Blue represents a low expression level, and red represents a high expression level.

The withering process significantly altered the levels of catechins and other flavonoids in tea leaves, through the expression of DEGs involved in catechin biosynthesis in at least one of the comparisons, as displayed in a heat map (Figure 5). The significantly downregulated expression levels of multiple DEGs were related to the biosynthesis of flavonols and catechins (Figure 5). Two genes associated with the biosynthesis of (+)-catechin (C), encoding anthocyanidin reductase (ANR) and leucoanthocyanidin reductase (LAR), as well as epigallocatechin (EGC), were significantly repressed by withering, and the amounts of C and EGC also decreased at the W4 stage. Furthermore, the DEGs involved in the upstream pathway of flavonol biosynthesis, such as 4-coumarate-CoA-ligase (4CL), chalcone synthase (CHS), and chalcone isomerase (CHI), were also significantly downregulated during withering. These results highlight that withering improved the palatability of tea leaves by moderately reducing the amounts of flavonol glycosides and catechins.

**Figure 6:**
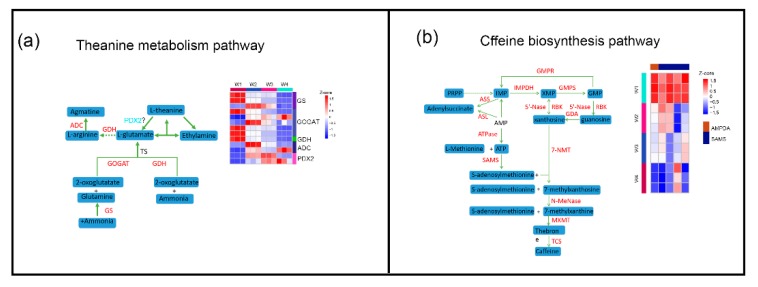
Effect of withering on theanine metabolism and caffeine biosynthesis pathways in tea leaves. A simplified model of theanine metabolism (**a**: left) and caffeine biosynthesis pathways (**b**: left) in tea plants and a heat map showing the expression pattern (**a**, **b**: right) of related DEGs based on the Z-core value at different timepoints during the withering process. The DEGs were strictly filtrated by |log2 (fold change)| ≥ 2 and *p* value < 0.05, with at least one of three comparisons (W2 vs. W1, W3 vs. W1 and W4 vs. W1). Blue represents a low expression level, and red represents a high expression level.

We found that theanine (γ-glutamylethylamide) was the most abundant free amino acid in tea (Figure 2b), which is consistent with a previous report [25]. Theanine, a major nitrogen reservoir in tea plants, decreased gradually during withering from the W1 to W4 stage (Figure 2b, Figure S2a). The DEGs in theanine biosynthesis were detected, and two glutamine synthetases (GSs), glutamate synthase (GOGAT) and glutamate dehydrogenase (GDH). were significantly downregulated at 24 h (Figure 6a). The theanine content was reduced, suggesting the theanine catabolic pathway plays a major role during the withering process. To date, little is known about the theanine catabolic pathway. Recently, a study found that theanine can be transformed into 2,5-dimethylpyrazine during tea manufacturing processes [26]. In addition, theanine from tea leaves was hydrolyzed into ethylamine and Glu using theanine hydrolases [27]. Theanine hydrolase might be homologous to Gln hydrolase (glutaminase) and PDX2 from *Arabidopsis* and have a glutaminase function [28]. In this work, two PDX2 homologs (CSS008524 and CSS034324) were predicted, and their expression levels gradually increased (Figure 6a), which was the opposite pattern to that of the changes of the theanine content. Therefore, we put forward the hypothesis that the PDX2 in tea leaves may promote the hydrolysis of theanine. The correlative molecular mechanism needs further investigation.

Caffeine, a vital metabolite for tea quality, is used to energize and promote briskness. Previous reports have shown that caffeine synthesis is independent of cacao and coffee lineages, which may be true in tea [29]. In addition, a S-adenosylmethionine synthetase (SAMS) and four AMP deaminases (AMPDAs) were significantly downregulated at the W4 stage (Figure 6b), which was similar to the trend for caffeine content.

To validate the reliability of the RNA-seq data, a total of 10 key DEGs (involved in the metabolism of critical components) were selected for qRT-PCR (Figure S5, Table S6). The expression profiles of all selected genes in RNA-seq and qRT-PCR were consistent, which confirmed that the genes generated from the RNA-seq data are trustworthy and reliable.

## 3. Materials and Methods

### 3.1. Plant Materials

Tea samples (one bud and two leaves) were harvested from *C. sinensis* (L.) (cv. Longjing 43), planted at the Tea Research Institute, Hangzhou Academy of Agricultural Sciences, Hangzhou, China (N30°11′7.21″, E120°03′52.45″) on 15 September 2019. To imitate the process of withering, the harvested tea leaves were placed into an artificial climate chest for 24 h under the conditions of 25 °C, 2000 lux, and 65% for the temperature, illumination intensity, and humidity, respectively. The amounts of phytochemicals in the tea leaves were determined at different timepoints (0, 3, 6, 9, 12, 15, 18, 21, and 24 h) during withering. Additionally, four samples of tea leaves withered for 0 (W1), 9 (W2), 15 (W3), and 24 h (W4) were used for transcriptome analysis.

The samples were fixed with liquid nitrogen immediately, and those for phytochemical determination were lyophilized and ground into 40-mesh with a Wiley mill. Three biological replicates were assayed for each group.

### 3.2. Measuring Endogenous Salicylic Acid, Jasmonic Acid, and Abscisic Acid

The extraction of hormones JA, SA, and ABA from tea leaves was based on a previously described method [30] with modifications. Briefly, we added 1 mL ethyl acetate, three kinds of internal hormone standards (D5-JA, D4-SA, and D6-ABA), and 0.1 g of crushed tea samples into tubes, shook them at 4 °C and 200 rpm in the dark overnight, then centrifuged them at 12,000 rpm and 4 °C for 10 min. The supernatant was transferred into a fresh 10 mL centrifuge tube. We used 1 mL of ethyl acetate to treat the residue, with 2 h of shaking and centrifugation at 12,000 rpm and 4 °C for 10 min. Finally, the combined supernatants were dried using a nitrogen blower. Subsequently, 0.5 mL of 70% chromatographic methanol (*v/v*) and the supernatant were mixed and filtered with a 0.22 μm filter. We used 200 μL of filtrate to analyze the concentrations of endogenous hormones, employing an LC–MS/MS instrument (Agilent 1260–6460), with an Agilent C18 column (150 mm × 2.1 mm, 3.5 μm). The mobile phases A and B were 0.1% formic acid and methanol, respectively. We performed a gradient elution, with a flow rate of 0.3 mL/min, using 40% B (8 min), 100% B (2 min), and 40% B (2 min). For MS detection, an electrospray ionization (ESI) source with negative ion multiple-reaction monitoring (MRM) mode was used.

### 3.3. Determination of Catechins and Purine Alkaloids

We extracted catechins and purine alkaloids from tea leaves using 80% methanol, according to a previous report [31,32]. An Agilent HPLC (Agilent Technology, San Diego, CA, USA), equipped with an Agilent-TC-C18 column (250 mm × 4.6 mm, 5.0 μm), was used to determine the catechin and purine alkaloid amounts. The column temperature was 25 °C, and the detection wavelength was 278 nm. The injection volume and column temperature were set to 2.0 μL and 35 °C, respectively. Mobile phase A consisted of 9% acetonitrile, 2% acetic acid, 89% water, and 20 mg/mL ethylenediamine tetraacetic acid, and solvent B consisted of 80% acetonitrile, 2% acetic acid, 18% water, and 20 mg/mL ethylenediamine tetraacetic acid. The flow rate was 1.0 mL/min of the mobile phase, and the detecting wavelength was 278 nm. The gradient elution was 100% solution A (10 min), 68% solution A, and 32% solution B (15 min) in the linear model, which was then held for 10 min. The retention times and peaks of samples were compared with those of authentic standards to identify and quantify individual catechins and purine alkaloids. The % dry weight of the tea samples was used to express the amounts. Standards of theobromine (TB), theophylline (TP), caffeine, and catechins were purchased from Sigma-Aldrich Chemical Reagent Co., Ltd. (Sigma-Aldrich, St. Louis, MO, USA). All chemical reagents were purchased from Sinopharm Chemical Reagent Co., Ltd. (Shanghai, China).

### 3.4. Determination of Free Amino Acid Content

Tea sample (0.15 g) and distilled water (25 mL) were mixed in a tube, which was placed in 100 °C water for 45 min and filtered and cooled to room temperature. Then, 25 mL of distilled water was used to dissolve the filtrate. Free amino acids were determined using HPLC (Agilent Technology, San Diego, CA, USA), with an ASB C18 analytical column (250 mm × 4.6 mm, 5 μm) and the parameters of column temperature were 30 °C with an injection volume of 2.0 μL. Mobile phase A contained 97% sodium acetate (0.1 M) and 3% acetonitrile (PH 6.5). Solvent B contained 80% acetic acid and 20% water, a mobile phase flow rate of 1.0 mL/min, and a detection wavelength of 254 nm. The gradient conditions were as follows: 93% solution A (5 min), 62% solution A and 38% solution B (25 min) in the linear model, which were changed to 100% solution B from 30 to 40 min, then to 7% solution B (5 min) and held for 10 min. The retention time and peak of samples and authentic standards were compared to identify and quantify the individual amino acids. The % dry weight of the tea samples was used to express the amounts. All chemical reagents were purchased from Sinopharm Chemical Reagent Co., Ltd. (Shanghai, China). Standards of free amino acids were purchased from Sigma-Aldrich Chemical Reagent Co., Ltd. (Sigma-Aldrich, St. Louis, MO, USA).

### 3.5. RNA Extraction, Illumina Sequencing, and Transcriptome Data Processing

The total RNA was extracted from the tea leaves according to the Thermo GeneJET Plant RNA Purification Mini Kit (Thermo Fisher Scientific Inc, USA). The quality of the RNA was examined using an Agilent Bioanalyzer RNA Nano chip Bioanalyzer (Agilent Technologies). After the poly(A) and rRNA were depleted, we purified mRNA and fragmented it using Fragmentation Buffer (Thermo Fisher Scientific Inc, USA). Random hexamer primers were used to perform reverse transcription of the RNA fragments. This was followed by second-strand cDNA synthesis with DNA polymerase I, dNTPs, and RNase H. Finally, cDNA libraries were created by amplifying the products, which were purified with QIAquick PCR extraction kit after end repair, A-tailing, and indexing ligation. We used a Qubit 2.0 DNA Broad Range Assay (Invitrogen, USA) to quantify the synthesized cDNA. An Illumina HiSeq 4000 Platform (Illumina, USA) was used to sequence the RNA libraries of the tea, and 2 × 150 bp paired-end sequencing reads were generated. FASTQC (version 0.10.1) (www.bioinformatics.bbsrc.ac.uk/projects/fastqc.) was used to assess the quality of the raw sequence data. Trimmomatic [33] was used to filter low-quality reads. The previously published genome database, CSS (cv. Shuchazao), was used as a reference genome to map the high-quality reads, using HISAT (v 2.0.5) with the default parameters. Stringtie (v 1.3.1) was used to assemble the aligned reads for all samples into transcripts [34]. We used StringTie-Merge to combine the StringTie assembly results of each sample, and the gene expression (FPKM) was calculated using StringTie’s own script, prepDE.py. The differently expressed genes (DEGs) were identified using Ballgown.

### 3.6. KEGG Pathway Enrichment Analyses

Kyoto Encyclopedia of Genes and Genomes (KEGG) is the main public database for pathways. Significant enrichment analysis of pathways uses the KEGG pathway as a unit to apply hypergeometric tests to find pathways that are significantly enriched in significant DEGs compared with the entire genomic background. The calculation formula is as follows [35]:

P=1−∑i=0m−1(Mi)(N−Mn−i)(Nn)

N: the total number of genes; n: the number of differentially expressed genes in N; M: the number of genes, annotated as a particular pathway; M: the number of differentially expressed genes, annotated as a particular pathway.

### 3.7. Co-Expression Networks

The R package was used to construct the co-expression networks [36] with all available genes, where the average FPKM of all samples was greater than 5 in all samples, and the parameters were defined. The substances of different tea samples that were significantly changed were used as traits to create a co-expression network. The satisfactory soft power threshold was determined to be 17. According to the soft power threshold, adjacency matrices were built using the adjacency function. According to the adjacency matrix, the topological overlap matrix (TOM) similarity algorithm was used to generate a topological overlap (TO) matrix, and TO similarity was used to hierarchically cluster the genes. The hierarchal clustering dendrogram was cut using the dynamic tree-cutting algorithm, and after decomposing/combining branches, a stable number of clusters to define the modules was reached. The PCA was used to calculate the summary profile in each module (module eigengene, ME). Next, we obtained the modules that had a higher TO value (average TO for all the genes in a given module) than the modules comprised of randomly selected genes.

## 4. Conclusions

In this study, we examined the amounts of hormones and critical components in tea leaves during the withering process and found that the dramatic alterations in the levels of abscisic acid (ABA), jasmonic acid (JA), and salicylic acid (SA), catechins, theanine, and caffeine were induced by withering. Compared with 0 h, many DEGs were identified at 9, 15, and 24 h, showing that significant changes in the transcriptome of tea leaves were caused by the withering process. WGCNA analysis of co-expression genes and substances indicated that ABA may be correlated with theanine metabolism during the withering process.

## Supplementary Materials

Supplementary materials can be found at https://www.mdpi.com/2223-7747/9/2/204/s1. Figure S1: Changes in the amounts of catechins (C, GC, EC, EGC, GCG, EGCG, ECG, and CG). Labeled points (not connected by the same letter) are significantly different at *p* values < 0.05, according to Duncan’s test. C, catechin; CG, catechin gallate; EC, epicatechin; ECG, epicatechin gallate; EGC, epigallocatechin; GC, gallocatechin; GCG, gallocatechin gallate; ECGC, epigallocatechin gallate. Figure S2: Changes in the content of caffeine and theanine. Labeled points (not connected by the same letter) are significantly different at *p* values < 0.05 according to Duncan’s test. Figure S3: Pearson correlation coefficient between hormones (SA, JA, and ABA) and critical components (catechins, theanine, and caffeine). Figure S4: Co-expression network analysis. WGCNA analysis of co-expressed genes and critical components. Each row corresponds to a module. The color and number of each cell at the row–column intersection indicates the correlation coefficient between the module and metabolites. A high degree of correlation between a specific module and the tissue type is indicated in red. Figure S5: Validation of the expression of candidate genes by qRT-PCR. Gene expression levels were determined by qRT-PCR and are presented as mean ± SD values, calculated by the 2^ΔΔCt^ method. Table S1: Statistics of the transcriptome data. Table S2: Expressed profiles and descriptions of all known genes. Table S3: Differentially expressed genes (DEGs) in the comparisons, W2 vs. W1, W3 vs. W1, and W4 vs. W1. Table S4: KEGG enrichment analysis of DEGs in W2 vs. W1, W3 vs. W1, and W4 vs. W1. Table S5: KEGG analysis of co-expressed genes in each high correlation module. Table S6: Details of the genes selected for validating the reliability of the RNA-seq data by qRT-PCR.
