# Supplementary figures and images for: Transcriptome and Phytochemical Analysis Reveals the Alteration of Plant Hormones, Characteristic Metabolites, and Related Gene Expression in Tea (Camellia sinensis L.) Leaves During Withering
Source: Plants (Basel). 2020 Feb 6;9(2):204. doi: 10.3390/plants9020204 (PMC7076645; doi:10.3390/plants9020204)

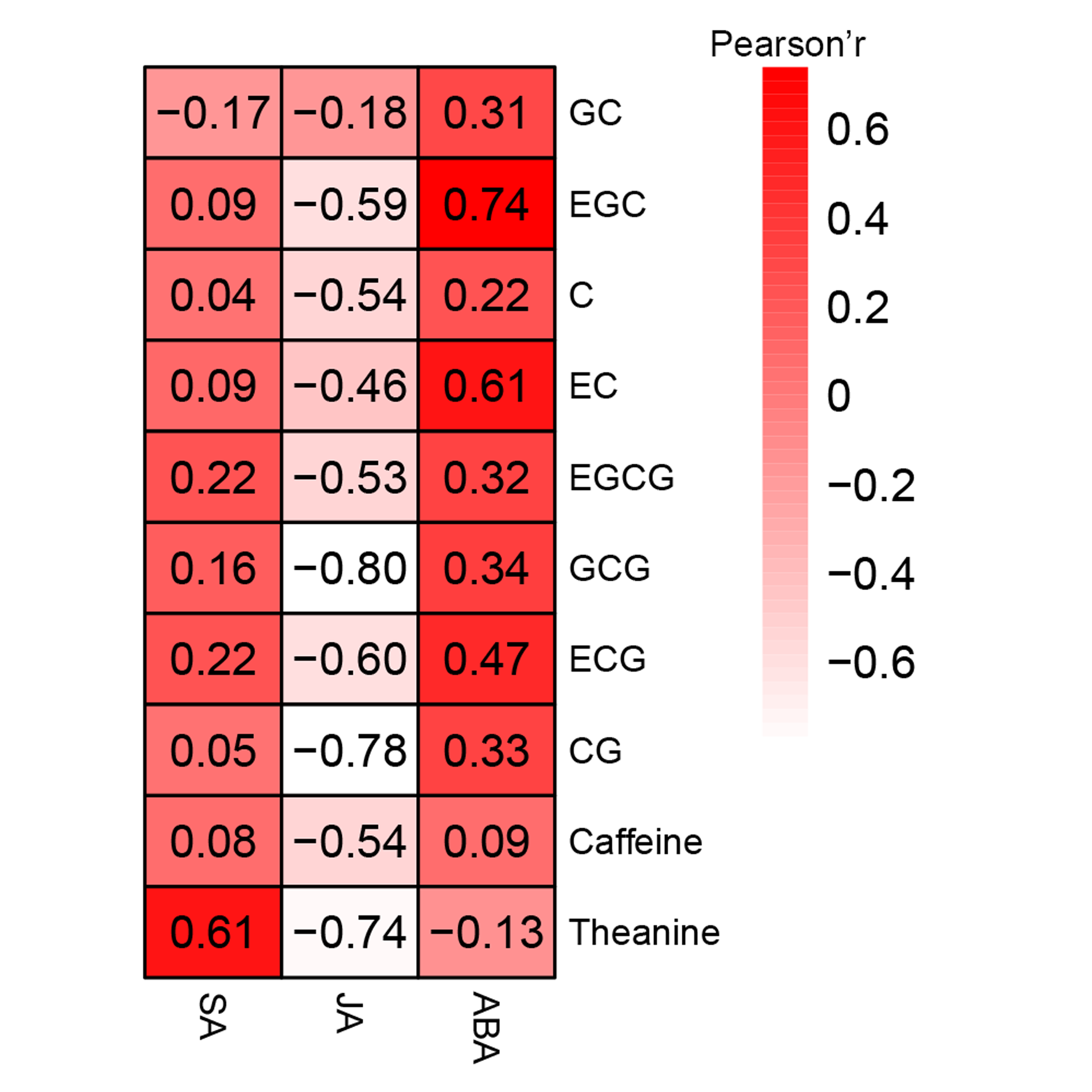

Supplement: Supplementary file 1 [file plants-09-00204-s001.zip › Supplementary materials/FigureS4.tif]

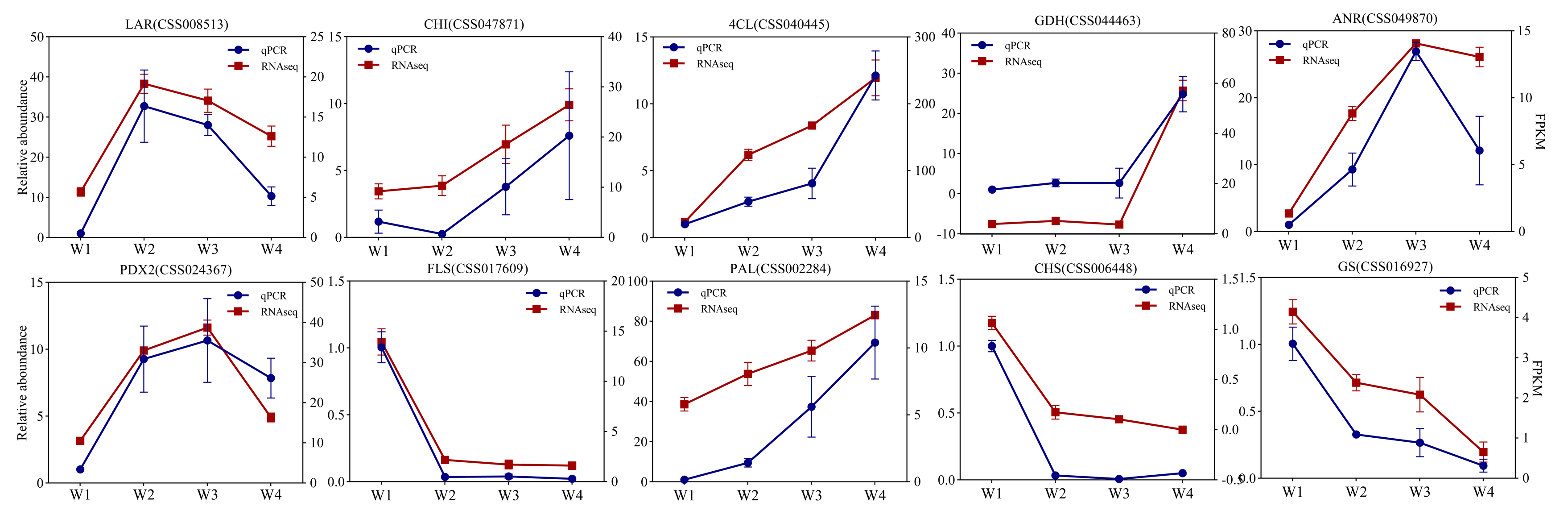

Supplement: Supplementary file 1 [file plants-09-00204-s001.zip › Supplementary materials/FigureS5.tif]

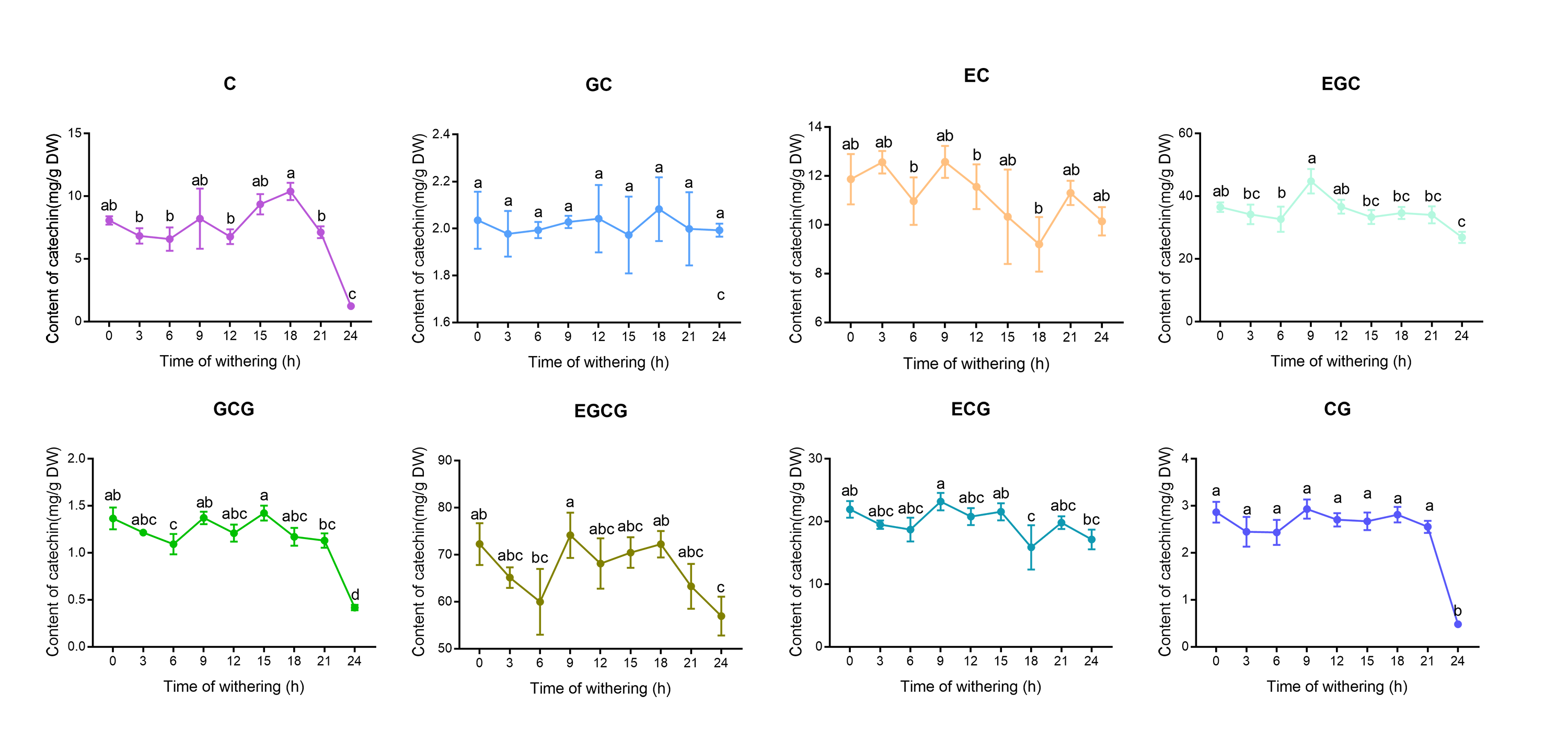

Supplement: Supplementary file 1 [file plants-09-00204-s001.zip › Supplementary materials/FigureS1.tif]

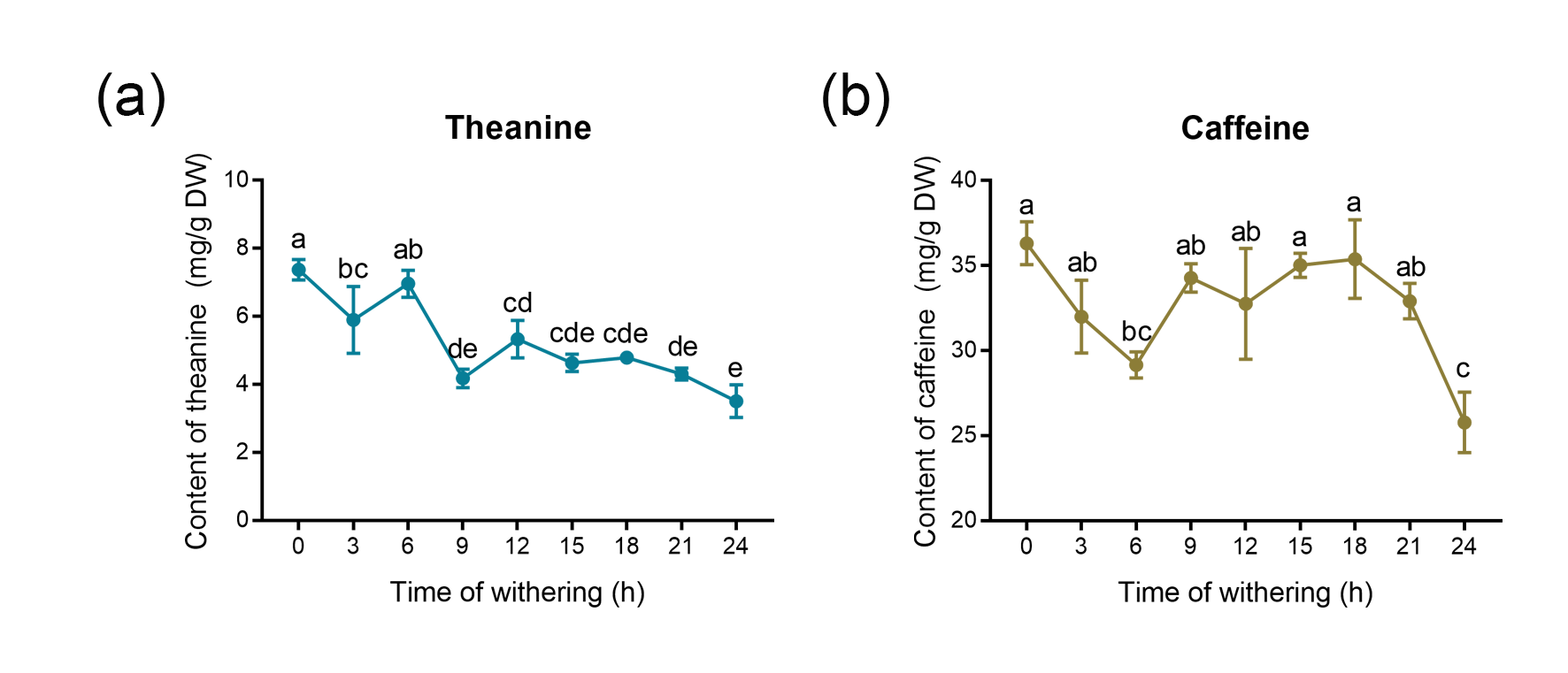

Supplement: Supplementary file 1 [file plants-09-00204-s001.zip › Supplementary materials/FigureS2.tif]

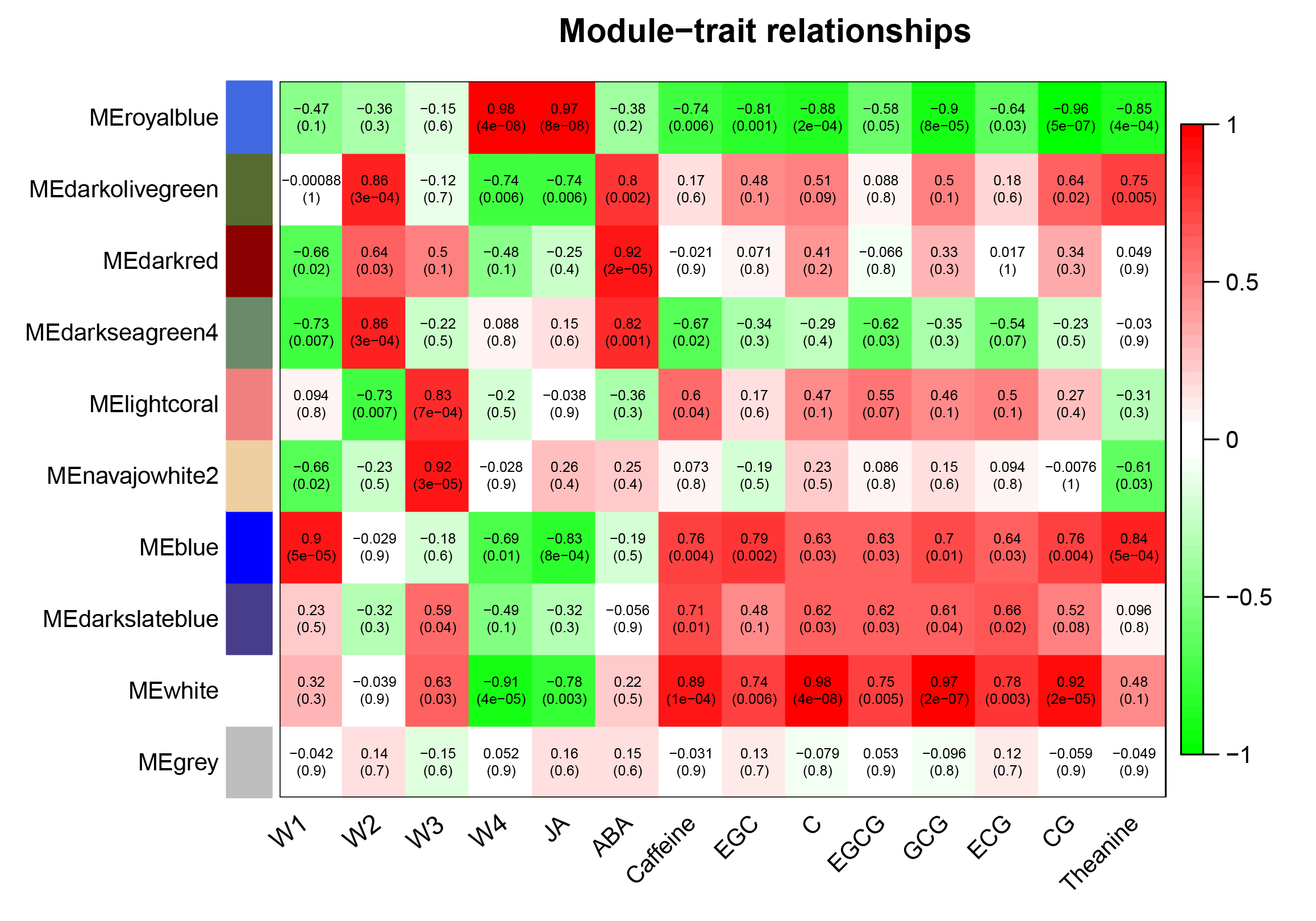

Supplement: Supplementary file 1 [file plants-09-00204-s001.zip › Supplementary materials/FigureS3.tif]
